# Using quantitative breath sound measurements to predict lung function following resection

**DOI:** 10.1186/1749-8090-5-81

**Published:** 2010-10-12

**Authors:** Rodolfo C Morice, Carlos A Jimenez, Georgie A Eapen, Reza J Mehran, Leendert Keus, David Ost

**Affiliations:** 1 Department of Pulmonary Medicine, The University of Texas MD Anderson Cancer Center, 1515 Holcombe Blvd. Unit 1462, Houston, Texas, 77030, USA; 2 Department Thoracic and Cardiovascular Surgery, The University of Texas MD Anderson Cancer Center, 1515 Holcombe Blvd. Unit 0445, Houston, Texas, 77030, USA

## Abstract

**Background:**

Predicting postoperative lung function is important for estimating the risk of complications and long-term disability after pulmonary resection. We investigated the capability of vibration response imaging (VRI) as an alternative to lung scintigraphy for prediction of postoperative lung function in patients with intrathoracic malignancies.

**Methods:**

Eighty-five patients with intrathoracic malignancies, considered candidates for lung resection, were prospectively studied. The projected postoperative (ppo) lung function was calculated using: perfusion scintigraphy, ventilation scintigraphy, and VRI. Two sets of assessments made: one for lobectomy and one for pneumonectomy. Clinical concordance was defined as both methods agreeing that either a patient was or was not a surgical candidate based on a ppoFEV_1_% and ppoDLCO% > 40%.

**Results:**

Limits of agreement between scintigraphy and VRI for ppo following lobectomy were -16.47% to 15.08% (mean difference = -0.70%;95%CI = -2.51% to 1.12%) and for pneumonectomy were -23.79% to 19.04% (mean difference = -2.38%;95%CI = -4.69% to -0.07%). Clinical concordance between VRI and scintigraphy was 73% for pneumonectomy and 98% for lobectomy. For patients who had surgery and postoperative lung function testing (*n *= 31), ppoFEV_1_% using scintigraphic methods correlated with measured postoperative values better than projections using VRI, (adjusted R^2 ^= 0.32 scintigraphy; 0.20 VRI), however the difference between methods failed to reach statistical significance. Limits of agreement between measured FEV_1_% postoperatively and ppoFEV_1_% based on perfusion scintigraphy were -16.86% to 23.73% (mean difference = 3.44%;95%CI = -0.29% to 7.16%); based on VRI were -19.56% to 28.99% (mean difference = 4.72%;95%CI = 0.27% to 9.17%).

**Conclusions:**

Further investigation of VRI as an alternative to lung scintigraphy for prediction of postoperative lung function is warranted.

## Background

Surgical lung resection remains the best option for cure of early stage non-small cell lung cancer and is the mainstay for treatment of other intrathoracic malignancies [1]. In assessing operability of patients with resectable lung malignancies, it is essential to define both the immediate perioperative risk and the long-term risk of pulmonary disability associated with loss of functional lung [1]. For patients with abnormalities on initial pulmonary function evaluation, quantitative radionuclide ventilation and perfusion studies are commonly used to evaluate split lung function and have been demonstrated to accurately predict postoperative lung function and outcome [2-5]. A projected postoperative FEV_1 _(ppoFEV_1_%) < 40% of predicted or a projected postoperative DLCO (ppoDLCO%) < 40% indicates an increased risk for perioperative death and cardiopulmonary complications with standard lung resection [5]. In a search for simpler alternatives to radionuclide tests for estimation of postoperative lung function, we studied quantitative measurements of acoustic vibratory energy at the chest wall generated by breath sounds during spontaneous breathing using a vibratory response imaging system (VRI).

In this pilot study, our primary objective was to assess the agreement of ppoFEV_1_% and ppoDLCO% as determined by VRI, perfusion, and ventilation scintigraphy. Our secondary objective was to obtain exploratory data comparing actual postoperative FEV_1 _values with ppoFEV_1_% values as determined by VRI or lung scintigraphy.

## Methods

### Study Population and Design

We prospectively studied patients with lung cancer or other intrathoracic malignancies, considered candidates for lung resection, who were referred to estimate postoperative lung function. The patients gave informed written consent to participate in the study. The protocol was approved by the Institutional Review Board of The University of Texas M.D. Anderson Cancer Center. All patients underwent lung function, radionuclide perfusion and ventilation scintigraphy, and VRI testing on the same day.

### Lung Function Testing

Pulmonary function tests were obtained according to published guidelines [6] utilizing a Pulmonary Function Laboratory 2400 System (SensorMedics; Anaheim, CA). Postoperative lung function was measured at 4-8 weeks after surgery with the same equipment.

### Radionuclide Perfusion and Ventilation Scintigraphy for Determining Regional Pulmonary Function

Radionuclide lung studies were performed using a multidetector system (Canberra Industries; Meriden, CT) according to the method described by Ali et al [7]. We considered the upper half of the tumor-bearing lung measurements to represent the functional loss after upper lobectomy, the lower half the functional loss for lower lobectomy (including the middle lobe on the right hemithorax), and the entire lung for pneumonectomy procedures.

### VRI for Determining Regional Pulmonary Function

**Figure 1:**
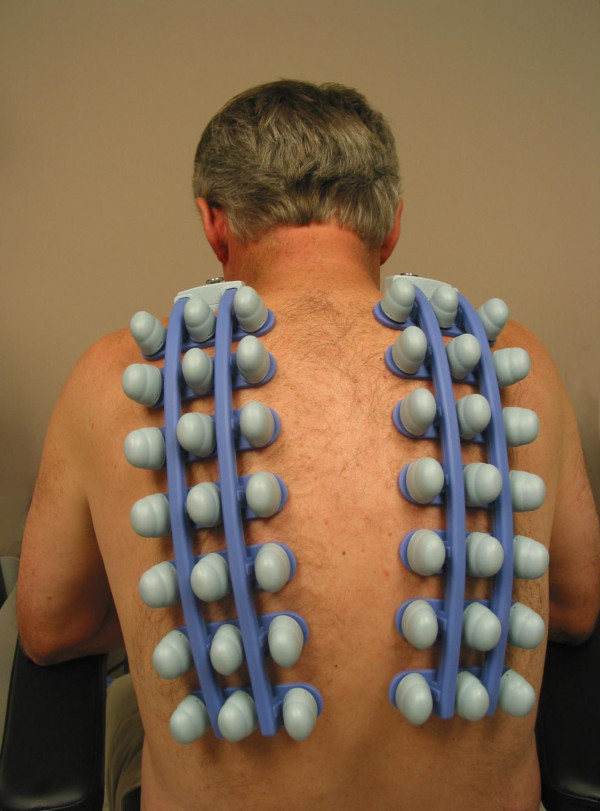
**Vibration response imaging system: the energy generated by the vibrations of the lungs during inspiration and expiration is discerned by two arrays of piezoelectric sensors during 12 seconds of recording**. Written informed consent was obtained from the patient for publication of the accompanying image.

Patients were tested using a VRIXP(tm) device (Deep Breeze(tm), Or-Akiva, Israel). Vibrations of the lungs were captured during inspiration and expiration via the mouth for 12 seconds by two arrays of seven or six piezoelectric sensors attached to the posterior chest by low vacuum (Figure 1). Signals were filtered, amplified, and converted into digital data for regional quantitative analysis based on location of each sensor [8,9]. Recordings with artifacts were excluded and two satisfactory recordings per patient were obtained. With the exception of recordings with artifacts, the second recording was always selected for analysis.

Similar to lung scintigraphy, vibrations originating from upper half of sensors in the tumor-bearing hemithorax represented the functional loss after upper lobectomy, the lower half the functional loss for lower lobectomy, and the entire sensor array for pneumonectomy procedures. An adjustment was made in which 5% of the total vibration energy on the left side was shifted to the right side (2% to the upper lung region and 3% to the lower) in order to compensate for greater lung sound distribution in the left lung as reported in the literature [10,11].

### Prediction of Postoperative Lung Function

Formulas for prediction of postoperative lung function were the same for VRI, ventilation, or perfusion, as follows [12]:

(1) ppoFEV_1_% (VRI, perfusion, or ventilation) = FEV_1pre-op _percent of predicted*(100%-projected percentage loss of lung function).

(2) ppoDLCO% (VRI, perfusion, or ventilation) = DLCO_pre-op _percent of predicted*(100%-projected percentage loss of lung function).

### Statistical Analysis

For the primary analysis, VRI was compared to perfusion scintigraphy. A separate analysis was performed comparing VRI with ventilation scintigraphy.

We used mean and standard deviation to describe continuous variables distributed normally. We used medians and interquartile ranges (25%-75%) for non-normally distributed data. We used paired T-tests to compare groups for normally distributed data and the Wilcoxon signed-rank test for non-normally distributed data. We assessed agreement between methods of determining projected percentage loss of lung function using a variety of methods. Our primary method was the Bland-Altman method [13]. We used Pittman's test of difference to evaluate correlation between differences between measures and the mean of the measures when performing Bland-Altman analysis [14].

We also performed simple and multivariable linear regression and used Pearson correlation coefficients to evaluate the strength of relationships between variables. For each test-VRI, perfusion, and ventilation - we used the following method to assess the ability of the test to explain the variability among the actual observed outcomes. The outcome we used was actual measured postoperative FEV_1_%. First, we assessed the relationship of baseline preoperative FEV_1_% with postoperative FEV_1_% using linear regression. Second, we assessed the relationship of residual functional lung as predicted by the testing method with postoperative FEV_1_% using linear regression. Residual functional lung was represented by the formula (100%-projected percentage loss of lung function). Third, we constructed a multivariable model consisting of baseline FEV_1_%, residual functional lung, and a variable representing the interaction of these two variables. Our fourth model used just the interaction variable. Note that this is what is used in standard clinical practice. We compared models using adjusted R^2 ^values. We used the methods of Cohen and Cohen to compare correlation coefficients from simple linear regression to determine which test was better at explaining the variance in measured postoperative FEV_1_[15]. We then performed the same analysis for the outcome of actual measured postoperative DLCO%. We also graphically analyzed regression results compared to the line of unity.

## Results

Ninety-nine patients (54 males and 45 females; age 65 ± 8 years, range 46-83 years) with: non-small cell carcinoma (n = 87), malignant pleural mesothelioma (n = 5), and intrapulmonary metastatic disease (n = 7) were entered in the study. Fourteen patients were excluded from the study due to protocol violation (n = 5) and technically inadequate VRI recordings (n = 9).

Evaluable data from 85 patients were included in the analysis. Baseline patients' characteristics are shown in Table 1. At time of data analysis, lung resections and postoperative pulmonary function tests had been obtained on 31 of these patients. Comparative analyses of predicted versus actual postoperative lung function measurements were based on 4 pneumonectomy and 27 lobectomy procedures.

**Table 1:** Baseline characteristics

Values	Variable
n = 85	All eligible patients
65 ± 8 yrs (range 47-83)	Age
M/F = 45/40	Gender
	Diagnosis
74	Non-small cell lung cancer
4	Malignant pleural mesothelioma
7	Metastatic disease to lung
	Baseline pulmonary function
79 ± 8	FEV_1_%
74 ± 22	DLCO%*
	Type of surgery performed**
6	Pneumonectomy
40	Lobectomy

* DLCO results were available on only 84 patients.

**Postoperative pulmonary function was available on only 31 patients (4 pneumonectomies, 27 lobectomies).

### Agreement between VRI and radionuclide studies for determining the projected percentage loss of lung function

**Figure 2:**
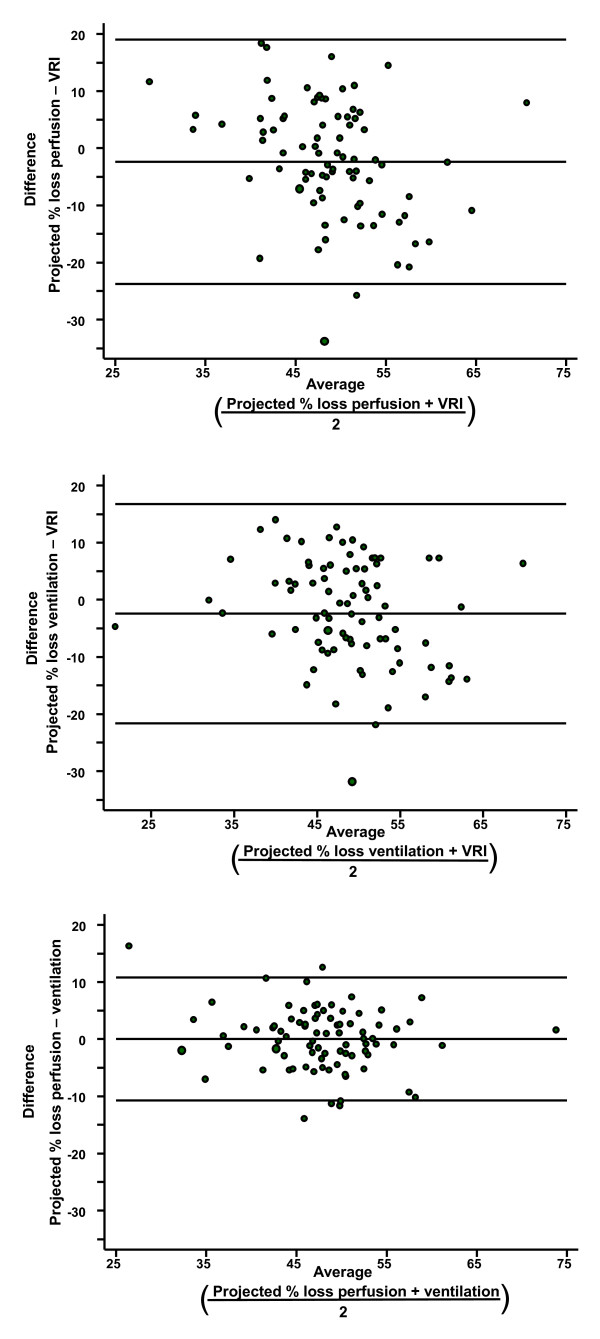
**Bland Altman plot for agreement of different technologies when calculating projected percentage of lung function loss with pneumonectomy**. (Top) Comparison of perfusion scintigraphy and VRI. (Middle) Comparison of ventilation scintigraphy and VRI. (Bottom) Comparison of perfusion scintigraphy and ventilation scintigraphy. Top and bottom horizontal lines represent limits of agreement; middle horizontal line is the mean difference.

**Figure 3:**
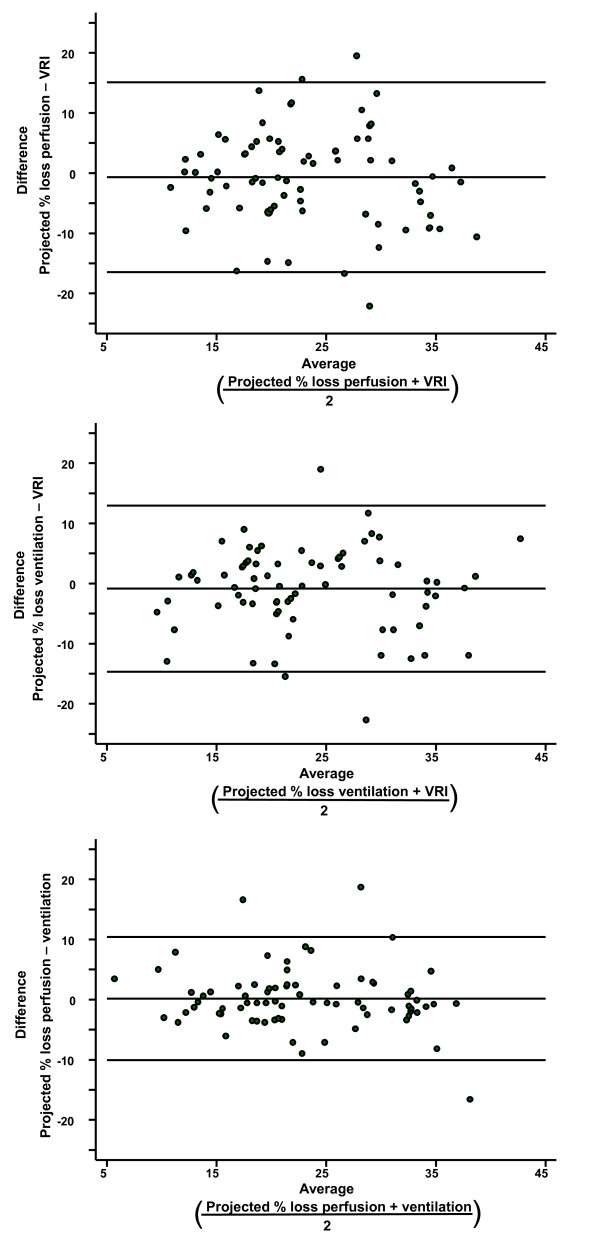
**Bland Altman plot for agreement of different technologies when calculating projected percentage of lung function loss with lobectomy**. (Top) Comparison of perfusion scintigraphy and VRI. (Middle) Comparison of ventilation scintigraphy and VRI. (Bottom) Comparison of perfusion scintigraphy and ventilation scintigraphy. Top and bottom horizontal lines represent limits of agreement; middle horizontal line is the mean difference.

Bland-Altman plots were used to calculate the agreement between the projected percentage loss of lung function for pneumonectomy (Figure 2) and lobectomy (Figure 3) estimated by VRI and radionuclide perfusion and ventilation tests. The limits of agreement are shown as two horizontal lines; the closer the lines are together, the better the agreement. The limits of agreement and mean difference are shown in Table 2 for each comparison. Agreement between radionuclide ventilation and perfusion was better than agreement between VRI and radionuclide perfusion (p < 0.0001).

**Table 2:** Agreement between methods for determining percentage of lung function lost

Mean Difference (95% CI)	Limits of Agreement	Comparison
		**Pneumonectomy**
2.38% (-4.69% to -0.07%)	-23.79% to 19.04%	Perfusion scintigraphy and VRI
-2.42% (-4.49% to -0.35%)	-21.61% to 16.78%	Ventilation scintigraphy and VRI
0.04% (-1.12% to 1.20%)	-10.72% to 10.79%	Perfusion and ventilation scintigraphy
		**Lobectomy**
-0.70% (CI -2.51% to 1.12%)	-16.47% to 15.08%	Perfusion scintigraphy and VRI
-0.86% (CI -2.45% to 0.73%)	-14.68% to 12.96%	Ventilation scintigraphy and VRI
0.16% (CI -1.02% to 1.34%)	-10.08% to 10.40%	Perfusion and ventilation scintigraphy

Agreement between ppoFEV_1_% as calculated by VRI and radionuclide perfusion and ventilation could not be performed, since these projections always used the same baseline preoperative FEV_1_% in their calculation (see methods, formula 1). This violates one of the fundamental assumptions of the Bland Altman method requiring that the two measures be independently taken. The same applies to agreement of ppoDLCO%.

### Clinical concordance between VRI and radionuclide studies

**Figure 4:**
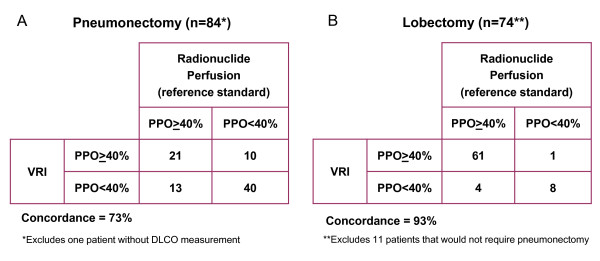
**Clinical concordance for predictions using VRI compared to predictions with perfusion scintigraphy for ppoFEV1% and ppoDLCO% > 40%**.

Since patients with ppoFEV_1_% and ppoDLCO% > 40% as predicted by perfusion studies are considered eligible for resection without need for further testing [1,12], we defined ppoFEV_1_% and ppoDLCO% values greater than or equal to 40% as positive (eligible for resection) and values below 40% as negative (further assessment needed). We defined clinical concordance for different testing methods (VRI, perfusion scintigraphy) as both methods agreeing that a patient either was eligible for resection (≥40%) or needed to undergo further assessment (< 40%). The clinical concordance for predictions using VRI compared to predictions with perfusion scintigraphy for ppoFEV_1_% and ppoDLCO% > 40% was 73% for possible pneumonectomy and 93% for possible lobectomy (Figure 4).

### Diagnostic test ability to explain variation in postoperative FEV_1_% and DLCO%

**Figure 5:**
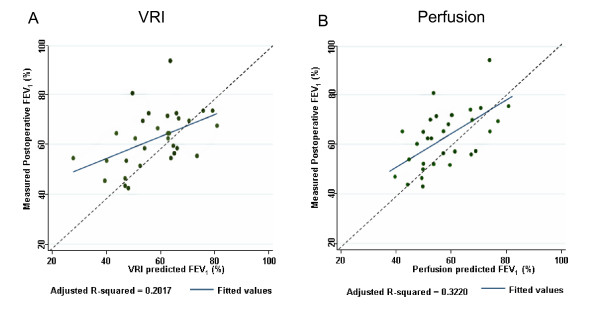
**Projected postoperative FEV1% compared to actual measurements**. (A) Projections based on VRI. (B) Projections based on radionuclide perfusion scans. Dotted line is the line of unity, indicating perfect agreement. Solid line is the regression line for least-squares fit.

Analyses of preoperative projections versus actual postoperative measurements are based on extent of surgery performed (4 pneumonectomy procedures and 27 lobectomy procedures) from 31 subjects who had surgery and postoperative lung function testing. The ppoFEV_1_% values calculated by VRI versus actual measurements of postoperative FEV_1_% are shown in Figure 5A. A similar comparison based on perfusion scintigraphy is shown in Figure 5B.

We further explored the ability of VRI, radionuclide perfusion and ventilation to explain variability in FEV_1_% measured postoperatively using multivariable models (Table 3). We used the adjusted R^2 ^as a measure of the proportion of the variability explained by the test. As expected, knowledge of baseline preoperative FEV_1_% was useful in explaining variation in FEV_1_% measured postoperatively (adjusted R^2 ^= 0.19). Estimating residual functional lung using perfusion scans, in the absence of knowing the baseline FEV_1_%, was not useful (adjusted R^2 ^= 0.02). Combining information of residual functional lung from perfusion scans with information about baseline FEV_1_% improved ability to explain variations in FEV_1_% measured postoperatively as compared to knowing just the baseline FEV_1_%. (p-value for the interaction term 0.02).

**Table 3:** Model fit as measured by adjusted R^2 ^for different test methods

Testing Method			Models
VRI	Ventilation	Perfusion	
0.19	0.19	0.19	Baseline FEV_1_%
-0.02	-0.02	0.02	Residual functional lung determined by test method
			Baseline FEV_1_% +
0.22	0.26	0.28	Residual functional lung determined by test method +
			(Baseline FEV_1_% × Residual functional lung by test method)
0.20	0.22	0.32	Baseline FEV_1_% × Residual functional lung by test method

We performed the same analysis for VRI. Again, knowledge of residual functional lung, in the absence of information about baseline FEV_1_%, was not useful. However, combining information of residual functional lung from VRI with information about baseline FEV_1_% did not significantly improve the ability to explain variations in measured postoperative FEV_1_% as compared to knowing just the baseline FEV_1_%.

The ability of radionuclide perfusion testing to explain variability in actual measured postoperative FEV_1_% was better than VRI, but the difference failed to reach statistical significance (adjusted R^2 ^0.32 for perfusion versus 0.20 for VRI; p = 0.32).

### Agreement between projected versus measured values of postoperative FEV_1_% and DLCO%

**Figure 6:**
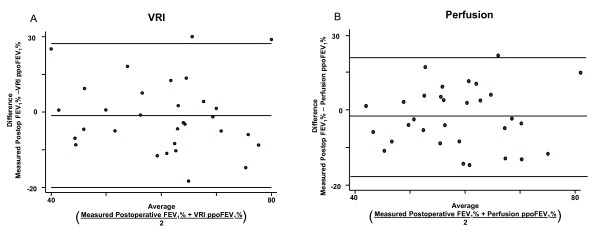
**Agreement between projected FEV1% and actually measured postoperative FEV1% for (A) perfusion and (B) VRI, as assessed by the Bland-Altman method**. Top and bottom horizontal lines represent limits of agreement; middle horizontal line is the mean difference.

**Figure 7:**
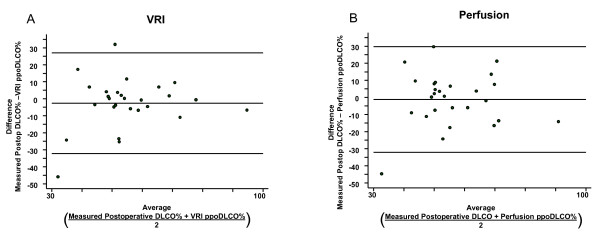
**Agreement between projected DLCO% and actually measured postoperative DLCO% for (A) perfusion and (B) VRI, as assessed by the Bland-Altman method**. Top and bottom horizontal lines represent limits of agreement; middle horizontal line is the mean difference.

Agreement between ppoFEV_1_% and measured postoperative FEV_1_% and DLCO% was assessed by the Bland-Altman method and shown in Figures 6 and 7. The limits of agreement and mean differences are given in Table 4. The agreement between measured postoperative FEV_1_% and ppoFEV_1_% using perfusion scintigraphy was not significantly different than the agreement between measured postoperative FEV_1_% and ppoFEV_1_% using VRI (p = 0.54). Similarly, the agreement between measured postoperative DLCO% and ppoDLCO% using perfusion scanning was not significantly different than the agreement between measured postoperative DLCO% and ppoDLCO% using VRI (p = 0.11).

**Table 4:** Limits of agreement and mean differences between projected and actually measured postoperative FEV_1_%. and DLCO%

Mean Difference (% of predicted)	Limits of Agreement (% of predicted)	Comparison
-3.44 (95% CI -7.16 to 0.29)	-23.73 to 16.86	Perfusion and actual FEV_1_%
-4.72 (95% CI -9.17 to -0.27)	-28.99 to 19.56	VRI and actual FEV_1_%
-1.16 (95% CI -7.26 to 4.94)	-32.01 to 29.69	Perfusion and actual DLCO%
-2.57 (95% CI -8.43 to 3.29)	-32.21 to 27.07	VRI and actual DLCO%

## Discussion

Our study describes the potential use of vibration response imaging (VRI) as a simpler alternative to lung scintigraphy for prediction of postoperative lung function in patients with intrathoracic malignancies. The question is whether the agreement between VRI and perfusion and between VRI and actual postoperative values is sufficient to consider using VRI in clinical practice. In this pilot study, we were able to obtain estimates of the limits of agreement between methods when calculating projected percentage of lung function lost. There was less agreement between VRI and perfusion than there was between ventilation and perfusion. To put this into context, when answering the question of surgical resectability, clinical concordance between VRI and perfusion was 73% for pneumonectomy and 93% for lobectomy. However, when comparing projected values to actual postoperative values, we failed to demonstrate a significant difference between VRI, perfusion, and ventilation. Yet, perfusion was able to explain more of the variability observed in postoperative FEV_1_% than VRI.

Many investigators have used the product-moment correlation coefficient (*r*) as an indicator of agreement. However, that is incorrect, since *r *measures the strength of a relation between variables but not agreement [13]. For example the series 2, 3, 4, 5, and 6 correlates well with the series 20, 30, 40, 50, and 60 but certainly they do not agree. It has been known for some time that a ppoFEV_1_% < 40% is an indicator of increased surgical risk [16,17]. For a new test to have clinical utility in predicting surgical risk, it is agreement with the existing standard, not correlation that is important. We compared agreement between techniques in terms of their projected percentage loss of lung function loss rather than ppoFEV_1_% or ppoDLCO%. It would have been incorrect to evaluate agreement between techniques in terms of their ppoFEV_1_% or ppoDLCO% using the Bland-Altman method. While this has been done by other investigators, it violates one of the key assumptions of the Bland Altman method, independence of measures, since all techniques are calculated values that share a common baseline number in the formula (either FEV_1_% or DLCO%).

We must emphasize that VRI measures acoustic energy, not lung perfusion or ventilation. While the mathematics of the calculation to arrive at projected percentage loss of lung function using VRI is analogous to quantitative lung scintigraphy, the physical properties being measured are distinctly different. The same could be said when comparing perfusion and ventilation - the mathematics is similar but the factors being measured are distinct. Hence, the proper term for comparison is not the calculated value of FEV_1_% or DLCO%, but the percentage of lung function lost as determined by vibration energy, perfusion, or ventilation.

In addition to measures of agreement, we were able to obtain further insights by performing longitudinal following up. We failed to demonstrate a significant difference between techniques in terms of their ability to estimate the actual observed postoperative FEV_1_% and DLCO%. We were able to demonstrate that combining information from perfusion scans with information about baseline FEV_1_% improved ability to explain variations in measured postoperative FEV_1_% as compared to knowing just the baseline FEV1% (p = 0.02). In contrast, we failed to demonstrate this for VRI and ventilation, although this may have been a function of the small sample size.

Clearly, a VRI study is simpler than other methods that have been used for estimation of postoperative lung function [18-21]. VRI testing can be performed by a trained technician and does not require administration of intravenous, inhaled, or external radiation. In spite of its relative simplicity, appropriate technical procedures are crucial. Recording artifacts arising from ambient noise or increased airway secretions should be avoided. Skin conditions or chest deformities may also interfere with the position and adhesion of sensors to the chest wall. During testing, attention should be placed to the quality, amplitude, and reproducibility of recordings. Interpretation of tests results must also consider clinical and radiographic correlations. Causes of discrepant results should be explored and an alternative method of testing should be considered in some cases.

## Conclusions

VRI technique would have the advantage of reducing overall costs in the process of preoperative evaluation and providing a non-invasive, complementary tool to pulmonary function testing within the scope of practice of the pulmonary technologist and the chest physician. However, additional studies are needed to determine if quantitative VRI could replace the radionuclide study.

## Abbreviations

CI: confidence interval; DLCO: diffusion capacity of the lung for carbon monoxide; F: female; FEV_1_: forced expiratory volume in 1 second; M: male; ppo: projected postoperative; VRI: vibratory response imaging system.

## Competing interests

The authors declare that they have no competing interests. The Department of Pulmonary Medicine of The University of Texas M.D. Anderson Cancer Center received funding from Deep Breeze Ltd. to conduct this study.

## Authors' contributions

RCM designed protocol, analyzed and interpreted the data, and prepared manuscript. CAJ contributed to study design, data analysis and interpretation, and preparation of manuscript. GAE contributed to data collection, patient entry into study, and preparation of manuscript. RJM contributed to interpretation of data and manuscript preparation. LK contributed to study design, patient entry and testing, and preparation of manuscript. DO designed statistical analysis, analyzed and interpreted data, contributed to preparation of manuscript. All authors read and approved the final manuscript.
